# Dopaminergic Progenitors Derived From Epiblast Stem Cells Function Similarly to Primary VM-Derived Progenitors When Transplanted Into a Parkinson’s Disease Model

**DOI:** 10.3389/fnins.2020.00312

**Published:** 2020-04-07

**Authors:** Sophie V. Precious, Gaynor A. Smith, Andreas Heuer, Ines Jaeger, Emma L. Lane, Stephen B. Dunnett, Meng Li, Claire M. Kelly, Anne E. Rosser

**Affiliations:** ^1^Brain Repair Group, School of Biosciences, Cardiff University, Cardiff, United Kingdom; ^2^School of Medicine, UK Dementia Research Institute, Cardiff University, Cardiff, United Kingdom; ^3^Behavioural Neuroscience Laboratory, Department of Experimental Medical Sciences, Lund University, Lund, Sweden; ^4^Stem Cell Neurogenesis Group, School of Medicine and Biosciences, Neuroscience and Mental Health Research Institute, Cardiff University, Cardiff, United Kingdom; ^5^School of Pharmacy and Pharmaceutical Sciences, Cardiff University, Cardiff, United Kingdom; ^6^School of Health Sciences, Cardiff Metropolitan University, Cardiff, United Kingdom; ^7^Wales Brain Repair and Intracranial Neurotherapeutics Unit, School of Medicine, Cardiff University, Cardiff, United Kingdom; ^8^MRC Centre for Neuropsychiatric Genetics and Genomics, School of Medicine, Cardiff University, Cardiff, United Kingdom

**Keywords:** transplantation, Parkinson’s disease, dopaminergic neurons, primary fetal ventral mesencephalon, stem cells

## Abstract

Neural transplantation in neurodegenerative diseases such as Parkinson’s disease (PD) offers to replace cells lost during the progression of the disease process. Primary fetal ventral mesencephalon (VM), the origin of *bona fide* midbrain dopaminergic (DAergic) precursors, is currently the gold standard source of cells for transplantation in PD. However, the use of tissue from this source raises ethical and logistical constraints necessitating the need for alternative supplies of donor cells. The requirement of any alternative donor cell source is to have the capability to generate authentic mature DAergic neurons, which could be utilized in cell-replacement strategies. Mouse pluripotent stem cells can efficiently generate electrochemically mature midbrain DAergic precursors *in vitro* using a stepwise control of FGF signaling. Here, we have compared DAergic transplants derived from two progenitor cell sources in an allograft system: mouse epiblast stem cells (EpiSC) and primary fetal mouse VM tissue. Cells were transplanted into the striatum of 6-OHDA lesioned mice pre-treated with L-DOPA. Drug-induced rotations, a number of motor tests and drug-induced abnormal involuntary movements (AIMs) were assessed. Functional improvements were demonstrated post-transplantation in some behavioral tests, with no difference in graft volume or the number of TH immuno-positive cells in the grafts of the two transplant groups. L-DOPA-induced AIMs and amphetamine-induced AIMs were observed in both transplant groups, with no differences in rate or severity between the two groups. Collectively, in this mouse-to-mouse allograft system, we report no significant differences in the functional ability between the gold standard primary VM derived and pluripotent stem cell-derived DAergic transplants.

## Introduction

In Parkinson’s disease (PD), neural transplantation of midbrain dopaminergic (DAergic) precursor cells aims to replace the nigral DAergic supply to the striatum which is lost during progression of this neurodegenerative disorder. Tissue taken from the region of the developing fetal brain where midbrain DAergic neurons originate, the ventral mesencephalon (VM), is currently regarded as the “gold standard” source of cells for clinical cell-replacement strategies in PD. Indeed, cells from primary fetal VM have been utilized in clinical trials of neural transplantation and have produced encouraging, albeit varied, results with respect to graft survival, and reinnervation within the host striatum and functional improvements (Lindvall et al., 1990, 1992; Mendez et al., 2005; Barker et al., 2013). Following reports of graft side effects, in particular graft-associated dyskinesias (Greene et al., 1999; Freed et al., 2001; Olanow et al., 2003; Barker et al., 2013) there was a pause in clinical fetal tissue transplants. Following extensive metanalysis and discussion of existing trials and further preclinical work (Barker et al., 2013, 2015; Parmar et al., 2019) the TRANSEURO trial commenced in 2012 (ClinicalTrials.gov NCT01898390). Results from the TRANSEURO trial are not expected until 2021 at the earliest (Barker and TRANSEURO consortium, 2019).

Animal models have provided much of the supporting data on transplant efficacy and the implantation of primary mouse VM into 6-OHDA lesioned mice has provided evidence of graft survival and integration with demonstration of functional improvements on various behavioral assessments (Shimizu et al., 1990; Thompson et al., 2009; Heuer et al., 2013; Kauhausen et al., 2013). The logistical issues surrounding the use of primary fetal tissue for therapeutic intervention in any disease render it complicated and impractical as a long-term prospective. Advances in the pluripotent stem cell research field have provided evidence that pluripotent stem cell-derived donor cells may ultimately serve as a valuable alternative source of cells for therapeutic application. Mouse pluripotent stem cells have been shown to have the capability to differentiate into DAergic neurons (Kim et al., 2002; Friling et al., 2009; Jaeger et al., 2011). Transplantation of mouse embryonic stem (ES) cell-derived DAergic precursors has resulted in survival and appropriate differentiation of a proportion of grafted cells, with a degree of rotational recovery (Kim et al., 2002; Rodriguez-Gomez et al., 2007; Battista et al., 2014).

With respect to human pluripotent stem cells, following application of systematic protocols DAergic precursor cells have been differentiated *in vitro* and *in vivo;* generating surviving grafts comprising DAergic neurons (Kriks et al., 2011; Grealish et al., 2014; Niclis et al., 2017). DAergic transplants derived from human ES cells have produced improvements in amphetamine-induced rotations (Kriks et al., 2011; Grealish et al., 2014; Niclis et al., 2017) and motor behaviors (Kriks et al., 2011). Of note, the study by Grealish et al. (2014) directly compared human primary fetal derived DAergic cells with human ES cell-derived DAergic cells in an immuno-deficient rat model of PD. They showed that transplanted human ES cells generated grafts with similar outgrowth, survival, and functional efficiencies to those generated from human fetal VM cells. Furthermore, using the modified rabies tracing system Grealish et al. (2015) have shown that human ES cell-derived DAergic grafts form reciprocal synaptic connections with host rat brain tissue.

One side effect of primary fetal dopamine transplants in PD is the development of graft induced dyskinesias (GIDs) in some patients (Freed et al., 2001; Hagell et al., 2002; Olanow et al., 2003). There is a literature reporting research into understanding the underlying causes and implications of graft-mediated abnormal movements so as to improve longitudinal outcomes following transplantation, with much mechanistic insight established using abnormal involuntary movements (AIMs) observed in the rat 6-OHDA lesion model (Carlsson et al., 2006; Lane et al., 2006, 2008, 2009a,b, 2010; Soderstrom et al., 2008, 2010; Steece-Collier et al., 2009; Lane and Smith, 2010; Tronci et al., 2015). These studies establish that L-DOPA-induced AIMs may change in the presence of the graft indicating early function, that amphetamine may induce abnormal movements which may be indicative of the potential for graft-induced dykinesia, but that true spontaneous dyskinesia have not been reliably observed. Previously, amphetamine induced AIMs have been identified in a mouse allograft paradigm in which primary mouse VM derived cells transplanted into the dopamine-depleted striatum resulted in development of AIMs similar to those seen in the rat model (Smith et al., 2012b).

Despite the increasing use of pluripotent stem cell-derived midbrain DAergic precursors for cell replacement strategies in animal models of PD, there is surprisingly limited literature directly comparing this relatively new cell source with the “gold standard” for neural transplantation that is primary fetal VM tissue. The majority of current studies are also confounded by the need for sustained immunosuppression and transplantation into xenogenic species (typically human cells to a rat host). Furthermore, there have been no studies comparing transplantation of these cells in the presence of pharmacological dopamine replacement strategies; medication that patients will have been on for many years pre-operatively, and which the majority will continue to be on post transplantation, albeit often at a lower dose. Direct comparison of efficacy post transplantation of pluripotent stem cell-derived grafts versus their fetal counterparts, should be made in order to ascertain more precisely how similarly or not DAergic precursors from the different cell sources behave post-transplantation. Here, we determined the efficiency of transplanted DAergic progenitor cells derived from two cell sources: bona fide primary mouse fetal VM tissue and mouse pluripotent stem cells that were differentiated toward a DAergic phenotype, to rescue deficits in the mouse 6-OHDA lesion model of PD. In addition, this head-to head comparison of authentic DAergic precursors with non-authentically derived DAergic precursors was carried out in an allograft system circumventing the potential impact of immunosuppression. Histological analyses were performed, and functional recovery was assessed looking at (1) drug-induced rotations; (2) a number of other motor tests of spontaneous behaviors; and (3) drug-induced dyskinesias (scoring AIMs). We found that, upon grafting, DAergic progenitors derived from the two donor cell sources were equipotent in all aspects of performance assessed.

## Materials and Methods

### Animals

All animal experiments were carried out in accordance with the UK Animals (Scientific Procedures) Act 1986 and approved by Local Ethics Review. Adult male mice (C57/Bl6) (Charles River, United Kingdom) were housed in groups of 4–6 in standard cages with a 12:12 h light:dark cycle and free access to food and water *ad libitum*. All procedures and testing were performed during the light phase.

### 6-OHDA Lesions and Transplantation

All surgical procedures were conducted under general anesthesia; induced with 2–3% isoflurane in oxygen and maintained at 1–2% isoflurane in a 2:1 oxygen/nitrous oxide mix. Post-operatively, mice were administered 0.5 ml saline solution containing 4% glucose, and Metacam (0.5 mg/kg), both via subcutaneous injection.

6-OHDA lesions were performed as previously described (Heuer et al., 2012; Smith et al., 2012a). Briefly, mice received unilateral 6-OHDA lesions to the medial forebrain bundle (MFB) at the following stereotaxic coordinates: AP = −1.2 mm, ML = −1.2 mm, and DV = −4.75 mm relative to bregma and the dura surface, with the incisor bar set at the interaural line. One microliter 6-OHDA was injected at a concentration of 6 μg/μl (in 0.2 mg/ml ascorbic acid in 0.9% saline) at a flow rate of 1 μl/min using a 30-gauge stainless steel cannula connected via fine polyethylene tubing to a Hamilton syringe on a microdrive pump, and followed by a 3 min post-infusion interval period where the cannula remained *in situ*.

Four to five weeks post-lesion, the lesion-induced deficit was assessed according to drug-induced rotations following administration of amphetamine (2.5 mg/kg; i.p.) and lesioned mice were group-matched according to rotational scores: a lesion only group and two transplant groups; primary VM and EpiSC. Mice in the two transplant groups received unilateral, intrastriatal transplants, ipsilateral to the lesion at the following stereotaxic coordinates: AP = +0.8 mm, ML = −1.7 mm, DV = −3.0 mm/−2.8 mm with the toothbar set at the interaural line. Two microliters cell suspension (150,000 cells/μl) were delivered using a Hamilton syringe, at 1 μl/min at each of the two heights. Following grafting, the needle was left at the graft site for a further two min before a slow withdrawal.

### Preparation of Cells for Transplantation

Primary fetal mouse tissue was obtained from C57/Bl6 female mice at embryonic day (E) 12. The VM from each embryo was dissected (based on Dunnett and Bjorklund, 1992) into Hanks balanced salt solution (HBSS) (Gibco), and a single-cell suspension was prepared. Briefly, HBSS was removed and tissue was incubated in trypsin (Worthington)/DNAse (Sigma) at 37°C for 10 min. Trypsin inhibitor (Sigma) was added and tissue was incubated at 37°C for a further 5 min. DMEM/F12 (Gibco) was added and cells were harvested by centrifugation at 1000 rpm for 3 min. The resulting pellet was resuspended in 200 μl DMEM/F12, triturated to produce a single-cell suspension and cells were counted using trypan blue exclusion. Cells were resuspended in DMEM/F12 at a density of 150,000 cells/μl for transplantation.

Mouse EpiSCs (EpiSC line Pitx3-LacZ) were differentiated according to Jaeger et al. (2011). Briefly, mouse EpiSCs were plated on fibronectin-coated plastics and cultured in N2B27 medium with bFGF (12 ng/ml, Peprotech) and activin A (20 ng/ml, R&D). When cells reached ∼65% confluency, this was designated as day 0 of monolayer differentiation. On day 0 cells were rinsed twice with phosphate-buffered saline (PBS) and cultured in retinol-free N2B27 medium with addition of PD0325901 (1 μM, Axon). The following day, half the medium was replaced with fresh medium. On day 2 cells were gently rinsed in PBS, mechanically dissociated in retinol-free N2B27 medium and seeded onto fresh fibronectin-coated plastic (1:3–6) in retinol-free N2B27 medium with SHH (200 ng/ml, C25 II-N, and R&D). Thereafter, medium was refreshed every other day. From day 5, FGF8b (100 ng/ml, Peprotech) and SHH (200 ng/ml) were added to the cultures. At day 7 of the differentiation protocol medium was removed, cells were washed in PBS and incubated in accutase (PAA Laboratories) at 37°C for 2–3 min. DMEM/F12 was added to the dish, cells were transferred to a 15 ml falcon tube and harvested by centrifugation at 1000 rpm for 3 min. The resulting pellet was resuspended in 200 μl DMEM/F12, triturated to produce a single-cell suspension and cells were counted using trypan blue exclusion. Cells were resuspended in DMEM/F12 at a density of 150,000 cells/μl for transplantation.

### Behavioral Analysis and Quantification

We assessed mice on a panel of behavioral tests previously described in the 6-OHDA lesion mouse model (Heuer et al., 2012; Smith et al., 2012a). Tests used here were balance beam, rotarod, rotation, and AIMs. The investigator was blind to which groups the mice had been assigned. Tests are described in brief here.

#### Spontaneous Rotations

Spontaneous rotations were measured as previously described (Heuer et al., 2012). The number of turns in the ipsilateral and contralateral direction were recorded and totaled. Data are represented as % of turns toward the contralateral direction.

#### Elevated Balance Beam

Elevated balance beam was performed as previously described (Heuer et al., 2012). Specifically, the time taken for the initial turn and beam traversal were measured.

#### Rotarod

Rotarod was performed and analyzed as previously described (Heuer et al., 2012). After training, mice were assessed for latency to fall from the rotating beam using an accelerating protocol.

#### Amphetamine-Induced Rotations

Amphetamine-induced rotations were assessed following administration of metamphetamine (2.5 mg/kg in 0.9% saline; i.p.). Mice were placed in glass beakers (as for the spontaneous rotations), and turns were measured using an automated rotometer system (Rotomax System, AccuScan Instruments Inc.) for a period of 90 min. Data are presented as average net rotations (ipsilateral minus contralateral) every minute and analyzed at both the 20 min and 70 min time bin.

#### Abnormal Involuntary Movements

Abnormal involuntary movements were scored following administration of L-DOPA (10 mg/kg, with 10 mg/kg benzerazide HCl in 0.9% saline; s.c.) and metamphetamine (2.5 mg/kg in 0.9% saline; i.p.). Mice were primed with daily L-DOPA injections (s.c) for 21 days prior to grafting to establish dyskinesia at baseline. Dyskinesia was equivalent in all lesioned groups prior to transplantation. L-Dopa induced dyskinesias (LIDs) were then scored again at 16 weeks post transplantation and AIMs were compared to the lesion only group. All animals were scored once every 20 min for 3 h.

The AIMs scoring criteria is based on the specific rating scales (Winkler et al., 2002; Cenci and Lundblad, 2007; Smith et al., 2012a, b). Duration and amplitude scores are the sum of all forelimb, hindlimb, orolingual and axial AIMs at all time points in the respective category. These are then in turn multiplied together to give a total integrated AIM score.

### Tissue Processing, Immunohistochemistry, Imaging, and Quantification

Mice were terminally anesthetized with sodium pentobarbital and transcardially perfused with approx. 30 ml PBS followed by 100 ml 1.5% paraformaldehyde (PFA) in PBS. Brains were removed, post-fixed for 24 h in 1.5% PFA, cryo-protected in 25% sucrose solution until they had sunk and were then sectioned coronally on a freezing-stage microtome at 40 μm thickness. Free-floating sections were processed for immunohistochemistry using the primary antibodies anti-tyrosine hydroxylase (TH; 1:1000; Chemicon) and anti 5-HT (1:15,000; Immunostar Inc.); biotinylated secondary antibodies; and ABC kit (Vectastain Elite) and diaminobenzidine (DAB; Vector Laboratories) for visualization, as previously described (Heuer et al., 2012). Immuno-labeled sections were visualized under a Leica DM/RBE light microscope and an Olympus BX50 light microscope with Visiopharm Integrator System software (version 4.4.6.9). For analysis of TH-fiber outgrowth, we adapted an existing protocol (Bagga et al., 2008). TH immunopositive projections were counted at 100 μm intervals from the periphery of the graft, vertically and horizontally, at dorsal, ventral, medial, and lateral aspects.

### Statistical Analysis

Data was analyzed using Graph Pad Prism 7 software or SPSS Version 25.0. When the full analysis revealed significant differences, pairwise comparisons between groups were undertaken using Bonferroni *post hoc* test with a *p* < 0.05 cut off for significance.

## Results

### Generation of Cells for Transplantation

**FIGURE 1:**
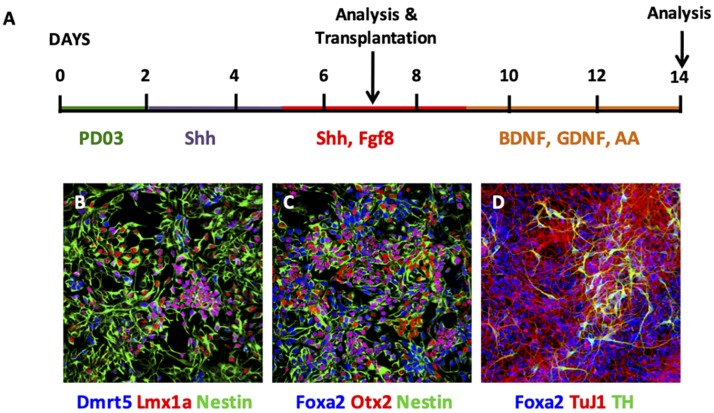
Characterisation of EpiSC monolayer differentiation. **(A)** Schematic of monolayer differentiation protocol. **(B–D)** Photomicrographs of immunofluorescent cytochemistry of cells at day 7 **(B,C)** and day 14 **(D)**. At day 7, EpiSCs expressed Dmrt5 (blue), Lmx1a (red), and Nestin (green) **(B)**; and Foxa2 (blue), Otx2 (red), and Nestin (green) **(C)**. At day 14, EpiSCs expressed Foxa2 (blue), TuJ1 (red), and TH (green) **(D)**. PD03, PD0325901; Shh, Sonic hedgehog; AA, ascorbic acid.

EpiSCs were cultured using a monolayer differentiation protocol (Figure 1A), where stepwise manipulation of FGF signaling was employed (Jaeger et al., 2011). Initially, PD0325901 was added for 2 days to block FGF signaling, followed by addition of Shh alone for 3 days and then from day 5 cells were exposed to both Shh and Fgf8. Analysis of cells at day 7 revealed expression of the neural precursor markers nestin and Otx2 (Figures 1B,C). Specifically, cells at this stage expressed Dmrt5, Lmx1a, and Foxa2 (Figures 1B,C). Further differentiation was undergone with continued exposure of cells to Shh and Fgf8, and subsequent addition of BDNF, GDNF, and ascorbic acid (AA) (Figure 1A). Cells analyzed at day 14 showed maintained expression of Foxa2, with expression of the neuronal marker TuJ1 and the DAergic neuronal marker TH (Figure 1D).

DAergic progenitors were taken at day 7 of the monolayer differentiation protocol and transplanted unilaterally into the dopamine depleted adult mouse striatum for comparison with primary VM derived DAergic progenitors via assessment of behavioral recovery and histological analysis.

### TH Expression in Grafts

**FIGURE 2:**
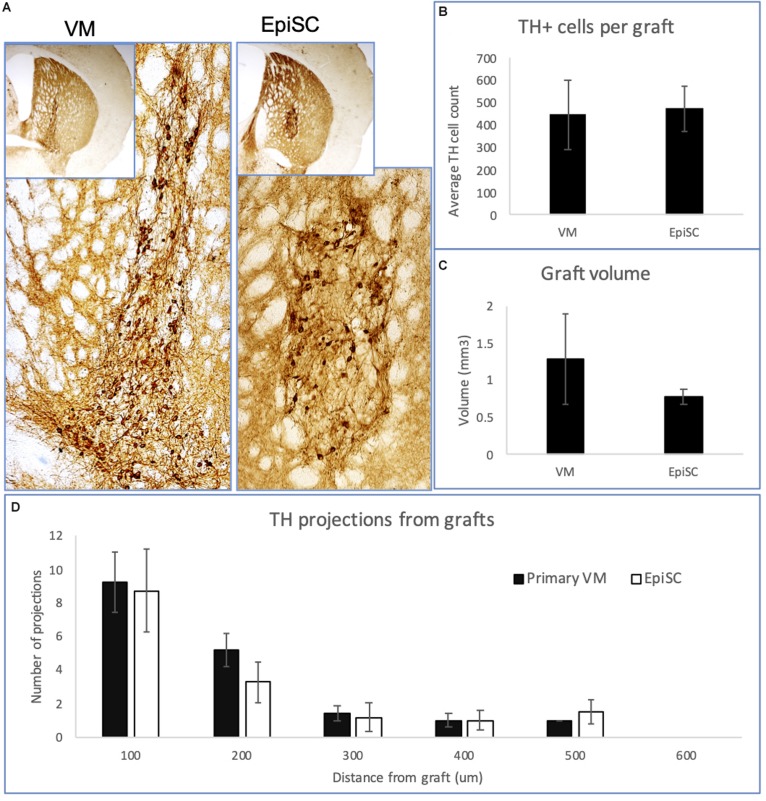
Expression of TH in transplants of VM and EpiSC derived DA progenitors at 16 weeks post-transplantation. **(A)** Photomicrographs of histological sections from mouse brains transplanted with VM derived DA progenitors and EpiSC derived DA progenitors, showing TH immunoreactivity. Higher magnification images reveal TH immune-positive cells within the grafts. Bar charts showing: **(B)** the average number of TH immuno-positive cells per graft; **(C)** the graft volume; and **(D)** the number and distance of TH projections. Error bars represent standard error of the mean.

DAergic precursors derived from primary VM and EpiSCs yielded TH immuno-positive cells up to 16 weeks post-transplantation (Figure 2A), with graft survival at 80% in both transplant groups. The number of TH immuno-positive cells per graft was similar in the two transplant groups (447 ± 154 for primary VM; and 474 ± 101 for EpiSCs) (*t*_23_ = 0.88, *n.s*) (Figure 2B). Additionally, graft volume, defined by the region of TH immune-staining, was not significantly different between the two transplant groups (*t*_23_ = 0.99, *n.s*) (Figure 2C). Moreover, there was no evidence of any graft overgrowth in transplants of both the primary VM or the differentiated EpiSC. Analysis of the morphology of the grafts revealed no significant differences in the number of TH positive projections extending from the grafts of the two transplant groups at any distance [*F*_(1,10)_ = 0.17, *n.s*] (Figure 2D). Grafts from both groups demonstrated that with increased distance from the graft the number of projections was lower [*F*_(4,40)_ = 27.92, *p* < 0.001].

Further analysis of the transplanted cells revealed a difference in the morphological subtype of the neurons between the two transplant groups [Chi-Square test: χ(2) = 7.636, *p* < 0.05]. Cells were assessed for the number of structures extending out of the soma: 36% of VM-derived grafted cells were unipolar, whereas 59% of EpiSC-derived grafted cells were unipolar; the number of bipolar neurons were similar with 39% from VM-derived and 36% from EpiSC derived grafted cells; and 25% of VM-derived grafted cells were multipolar, whereas 5% of EpiSC derived grafted cells were multipolar. Measurements of the cell soma diameter and area of TH immunopositive transplanted cells revealed differences between cells derived from the two transplant groups (soma diameter: primary VM 28.65 ± 0.61 μm; EpiSC derived 24.79 ± 0.72 μm (*t*_13_ = 3.79, *p* < 0.01); soma area: primary VM 153.92 ± 11.21 μm^2^; EpiSC derived 108.18 ± 6.75 μm^2^ (*t*_13_ = 3.73, *p* < 0.01).

The number of 5-HT immuno-positive cells per graft was low and highly variable. There was no significant difference between the two donor cell types (primary VM: 110.23 ± 91.86; EpiSC derived cells: 119.42 ± 55.35) (*t*_5_ = 0.09, *n.s*), although it is noteworthy that 5-HT immune-positive cells were only detected in one third of surviving primary VM derived grafts but were found in all surviving EpiSC derived grafts analyzed.

### Behavioral Analysis

In order to determine whether grafts left to mature for 16 weeks post-transplantation caused any functional recovery in hemi-parkinsonian mice, we performed several behavioral tests as previously defined in Heuer et al. (2012). Significant differences were observed between groups on the rotarod test [*F*_(__3,39__)_ = 5.64, *n.s*]; *post hoc* tests confirmed that 6-OHDA lesioned mice had a deficit on rotarod performance compared to unlesioned controls (*p* < 0.05) (Table 1). This deficit was not recovered in mice with primary VM transplants (*p* < 0.01), but was partially alleviated in mice with EpiSC transplants (*p* < 0.05) (Table 1). Mice with unilateral 6-OHDA lesions exhibit spontaneous rotational bias when placed in cylinders, caused by dopamine released from the intact side [*F*_(__3,39__)_ = 2.75, *p* < 0.01; *post hoc p* < 0.05]. This deficit was sustained in both of the transplant groups post-transplantation, with no significant difference observed when compared with the lesion-only mice; yet the two transplant groups were still significantly different from the un-lesioned controls (posthoc comparisons: primary VM: *p* < 0.05; EpiSC: *p* < 0.05) (Table 1). Further motor coordination assessments were made using the balance beam. No deficits were found in any of the groups in the time taken for mice to make the initial turn to face the correct direction to initiate the test. However, 6-OHDA lesioned mice showed an increased latency to traverse the beam compared to unlesioned controls [*F*_(__3,39__)_ = 4.16, *p* < 0.05; *post hoc p* < 0.05]. This deficit persisted in transplanted animals of both groups (primary VM: *p* < 0.05; EpiSC: *p* < 0.05) (Table 1).

**TABLE 1:** Assessment of behavioral tests.

	Un-lesioned control	Lesioned control	Primary VM	EpiSC
Rotarod (secs)^1^	135.4 ± 10.57	84.1 ± 12.7*	74.5 ± 12.5**	99.4 ± 10.5^#^
Spontaneous rot (% contralateral)^1^	48.9 ± 2.8	29.7 ± 8.9*	22.0 ± 9.1*	27.2 ± 7.8*
Beam traversal (latency)^1^	7.66 ± 0.42	12.7 ± 0.89*	16.17 ± 2.42*	15.65 ± 3.02*

Assessments performed at 16 weeks post-transplantation. ^1^Data is presented as mean ± SEM and was analyzed by one-way ANOVA using Tukey *post hoc* tests. Significance is annotated as ^∗^*p* < 0.05 and **p < 0.001 compared to un-lesioned controls and ^#^*p* < 0.05 compared to lesioned controls.

### Dopamine Neuron Transplantation Partially Rescues Amphetamine-Induced Rotations

**FIGURE 3:**
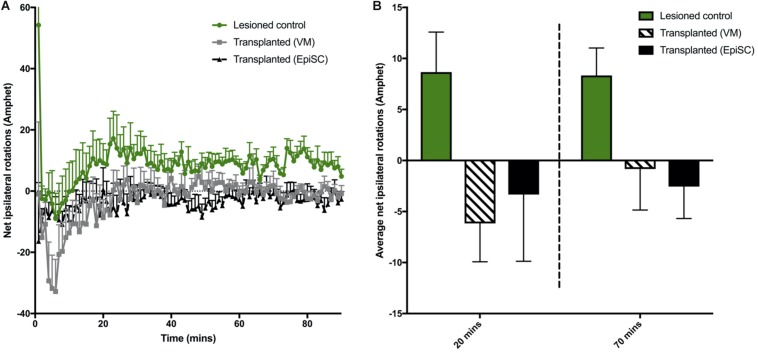
Assessment of drug induced rotations. **(A)** Total net rotations were recorded every min for 90 min following Amphetamine (Amphet) injection. **(B)** Average net rotations at both 20 and 70 min were increased in lesioned animals although not significantly, with little change in transplanted (VM) and transplanted (EpiSC) groups. Data was analyzed by 1-way ANOVA (*p* < 0.07 at 70 min). Graphs are expressed as Mean ± SEM and *N* = ≥ 6 mice per group.

A more pronounced rotational response can be observed in hemi-parkinsonian mice following administration of amphetamine. The 6-OHDA lesioned group displayed a higher net ipsilateral rotational response over 90 min compared to transplanted animals when the data were examined in 1 min bins (Figure 3A). However, there was no significant difference in the average net rotations at both 20 min [*F*_(__2,18__)_ = 1.74, *n.s*] and 70 min [*F*_(__2,18__)_ = 4.16, 0.1 > *p* > 0.05] compared to lesioned controls although a clear trend toward a reduction in ipsilateral rotations and a predominance of contralateral rotations was evident (Figure 3B). Taken together, these results suggest that both VM and EpiSC dopamine neuron transplants were capable of releasing dopamine when stimulated with amphetamine but had a minimal effect on functional recovery and the most sensitive tests for capturing modest effects were the rotarod and amphetamine-induced rotations.

### Dopamine Neuron Transplantation Did Not Suppress l-Dopa Induced Dyskinesia and Caused Graft-Induced Dyskinesia

**FIGURE 4:**
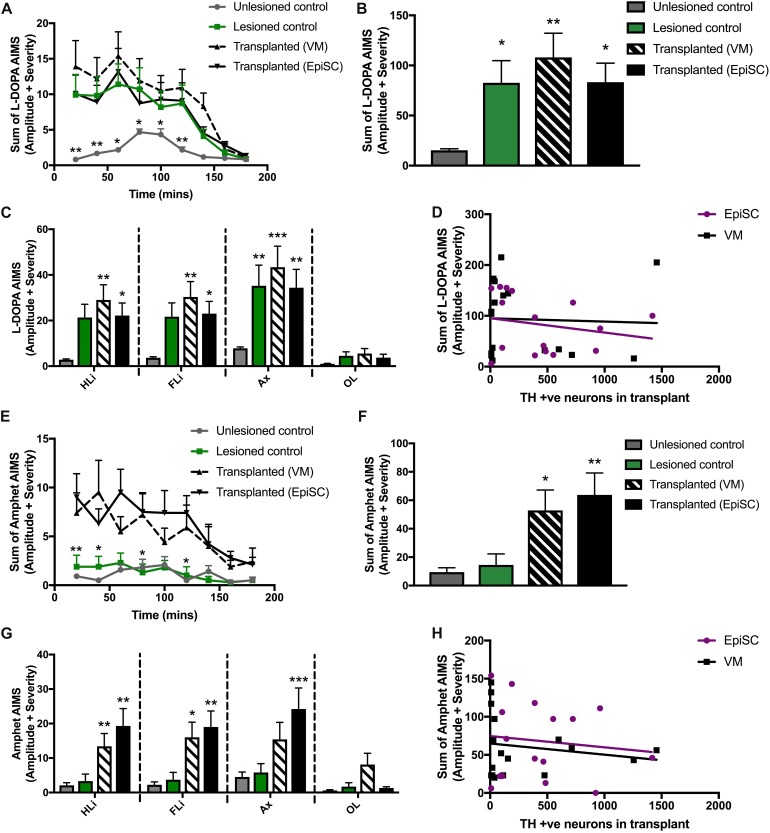
Assessment of abnormal involuntary movements (AIMS). **(A)** AIMS were recorded every 20 min following L-Dopa injection. **(B)** Average AIMS were significantly increased in lesioned, transplanted (VM), and transplanted (EpiSC) following L-Dopa. **(C)** L-Dopa induced AIMS of the hind limb (HLi), fore limb (FLi), and Axial (Ax) were increased in all experimental groups compared to control. **(D)** L-Dopa induced dyskinesia was not correlated by grafted TH +ve cell number. **(E)** AIMS were recorded every 20 min following amphetamine (Amphet) injection. **(F)** Average AIMS were significantly increased in lesioned, transplanted (VM), and transplanted (EpiSC) following Amphet. **(G)** Amphet induced AIMS of the hind limb (HLi), fore limb (FLi), and Axial (Ax) were significantly increased in all experimental groups compared to control. **(H)** Graft-induced dyskinesia was not correlated by grafted TH +ve cell number. Data was analyzed by 1-way ANOVA and significant differences annotated as *p < 0.05, **p < 0.01, and ***p < 0.01 compared to control. For A and E data was analyzed by MANOVA and significance annotated as *p < 0.05 and **p < 0.01 compared to control. Graphs are expressed as Mean ± SEM and *N* = ≥ 6 mice per group.

Mice were treated with L-DOPA for 3 weeks prior to transplantation (described in Smith et al., 2012a; Francardo and Cenci, 2014). At 16 weeks post-transplantation mice were challenged with a single injection of L-DOPA to determine if one cell type was more prone to provoke LIDs than the other by assessing AIMs. AIMs were observed in all lesioned mice (lesion only and lesion plus transplant) compared to unlesioned control mice from 20 to 140 min post-injection (Figure 4A). Significant differences between groups were observed [*F*_(__3,42__)_ = 4.70, *p* < 0.01]. Average AIMs were significantly increased in the 6-OHDA lesioned group compared to unlesioned controls as expected (*p* = 0.045), and were also elicited in both transplant groups: primary VM (*p* < 0.01) and EpiSC derived (*p* < 0.05) (Figure 4B). AIM subtypes were then analyzed separately and significant differences between the groups were observed [*F*_(__3,168__)_ = 14.22, *p* < 0.001]. L-DOPA induced AIMs of the hind limb were observed in VM (*p* < 0.01) and EpiSC (*p* < 0.05) transplanted mice, which were minimal in unlesioned controls (Figure 4C). This was also seen for forelimb AIM quantification (VM: *p* < 0.01; EpiSC: *p* < 0.05) (Figure 4C). L-DOPA induced AIMs were also observed in the axial category (lesioned: *p* < 0.001; VM: *p* < 0.001; EpiSC: *p* < 0.01), yet no orolingual AIMS were observed (lesioned: *p* = *n.s*; VM: *p* = *n.s*; EpiSC: *p* = *n.s*) (Figure 4C). AIMs were not correlated to the number of TH neurons in the grafts (VM: *p* = *n.s*; EpiSC: *p* = *n.s*) (Figure 4D).

Rodent recipients of DAergic transplants can also develop a form of GID when administered amphetamine. Whilst LID did not distinguish between lesion only and transplanted mice, hemi-parkinsonian mice receiving primary VM and EpiSC derived dopamine precursors showed clear amphetamine induced AIMs following administration of amphetamine which were not elicited in the unlesioned and lesion only control mice [*F*_(__3,42__)_ = 5.55, *p* < 0.01] (Figure 4E). Pairwise comparisons indicated that average AIMS were significantly greater following primary VM (*p* < 0.05) and EpiSC derived (*p* < 0.01) dopamine precursor transplantation (Figure 4F). Significant differences were also observed for amphetamine driven AIMs when quantified per anatomical subcategory [*F*_(__3,168__)_ = 16.05, *p* < 0.001]. Sterotyped movements of the hind limb were significantly increased in primary VM (*p* < 0.01) and EpiSC (*p* < 0.01) transplanted mice compared to unlesioned controls (Figure 4G). This increase was also seen in the forelimb (VM: *p* < 0.05 and EpiSC: *p* < 0.01), yet axial AIMs were only significantly increased by EpiSC derived dopamine precursor transplantation (*p* < 0.001) (VM: *p* = *n.s*) (Figure 4G). No significant levels of orolingual AIMs were observed in any treatment group (VM: *p* = *n.s*; EpiSC: *p* = *n.s*) (Figure 4G). Amphetamine stimulated AIMs were not correlated to the number of TH + ve neurons in the grafts (VM: *p* = *n.s*; EpiSC: *p* = *n.s*) (Figure 4H). These data suggest that GIDs may occur following transplantation regardless of dopamine neuron source.

## Discussion

Efficient generation of midbrain DAergic precursors *in vitro* has been shown by employment of a stepwise control of FGF signaling (Jaeger et al., 2011). Precursor cells, at day 7 of the differentiation protocol (the day that cells were harvested for transplantation in our study), expressed Dmrt5, Lmx1a, and Foxa2 which are characteristic markers of midbrain DAergic neural progenitors (Andersson et al., 2006; Ferri et al., 2007; Gennet et al., 2011). Mature neurons differentiated using this protocol expressed TH and showed functional neuron-like electrophysiological properties (Jaeger et al., 2011). Furthermore, following 14 days of differentiation, around 50% of neurons were TH positive, and of the TH-positive cells, around 70% were immunopositive for Pitx3, another marker of midbrain DAergic neurons (Jaeger et al., 2011). We investigated these cells *in vivo*, assessing their functional capability in a direct comparison to the current gold standard for DAergic transplants: authentic DAergic progenitors harvested from primary fetal VM.

Despite the fast-moving pace of the stem cell field in the progression of differentiation protocols for generation of specific neuronal phenotypes for research into neurodegenerative disease for disease-in-a-dish paradigms and cell replacement strategies (including DAergic neurons for PD), there is limited literature on comparisons of *in vivo* performance of stem cells with their primary fetal neural tissue counterparts. In particular, and of note in the PD model system, there is little utilization of the mouse model, which means that since rat pluripotent stem cells are not well-established, there is limited experience of transplantation in an allograft model system. Here, we carried out a direct comparison of allografts of primary fetal VM derived and EpiSC derived DAergic precursors transplanted into a hemi-parkinsonian mouse model. At 16 weeks post-transplantation we demonstrated surviving DAergic grafts with no sign of overgrowth or teratoma formation in either group. Our histological analyses showed no differences in the grafts yielded by the two transplant groups with respect to number of TH immuno-positive cells and graft volume. We also recorded no difference in the distance that TH positive cells projected outward from the grafts of both donor cell groups. These findings are consistent with those of Yurek and Fletcher-Turner (2004) who also demonstrated no difference in innervation of TH outgrowth in the striatum from DAergic grafts derived from both primary fetal VM and ES cells. In our analysis of the morphology of the graft-derived TH-positive cells we showed that the majority of cells in the EpiSC grafts were unipolar with only a small proportion of multipolar neurons. In contrast, graft-derived neurons from the primary VM transplant group were more evenly distributed between unipolar, bipolar, and multipolar subtypes. In addition, we identified a difference in the cell soma size between the two groups of grafted DAergic neurons. Specifically both the diameter and area of the cell soma were larger in the primary VM derived neurons compared with the EpiSC derived DAergic neurons. Differences in neuronal morphology may be important in the functionality of the graft, however, this warrants further investigation.

With respect to 5-HT, we identified a small population of 5-HT immuno-positive neurons within both VM and EpiSC grafts, present in similar proportions. In VM tissue transplants, 5-HT neurons are incorporated if there is a wider caudal dissection encompassing some of the developing raphe nuclei. The population of 5-HT neurons within a graft is of interest as there has been speculation about their role in graft-induced dyskinesia (Lane et al., 2009b; Politits et al., 2010; Politis et al., 2011), and although this has not been conclusively demonstrated to be a concern, protocols for future clinical trials are dedicated to generating specific DAergic lineages which in effect eliminate the risk 5-HT neurons may contribute (Barker et al., 2017). There is, however, no evidence that the 5-HT neurons contribute to the functional efficacy of the graft.

It is known that DAergic neurons in the transplant are necessary to achieve recovery on the amphetamine-rotation task (Dunnett et al., 1988) and functional improvement on this task directly corresponds to synaptic dopamine release. In addition, it is thought that *in vivo* performance of transplantation relies upon appropriate DAergic number, subtype and outgrowth into the dorsolateral striatum, reviewed in Peng and Copray (2016). This has been extensively studied following primary tissue transplants in rodents, but studies which show functional recovery with stem cell transplants can be limited, in particular in the mouse model. One previous study showed that mouse ES cells transplanted into 6-OHDA lesioned rats did not induce functional recovery compared to primary mouse fetal VM transplantation, despite yielding similar DA cell numbers and morphologies (Yurek and Fletcher-Turner, 2004). However, over recent years it has been suggested that ES cells can have comparable properties to primary tissue in immunocompromised rats, indicating that long term functional improvements are possible (Grealish et al., 2014). In addition, it has been shown that DA release measured through microdialysis is higher in rodents that had received DAergic transplants derived from ES cells compared with lesion only controls, corresponding to improved amphetamine-induced rotations (Rodriguez-Gomez et al., 2007). We demonstrate that functional stem cell grafts are possible in mice that are not immunosuppressed, demonstrated by enhanced rotarod performance, increased amphetamine-induced rotation and decreased L-DOPA-induced dyskinesia. However, further optimisation is needed to increase the beneficial effect in order to alleviate fine motor deficits on behavioral tests such as balance beam. ES cell differentiation protocols, transplantation procedures and animal model choices may therefore be key to induction of functional recovery.

In this direct comparison of transplantation of DAergic neurons derived from a pluripotent stem cell source with primary fetal DAergic progenitors from VM we used mouse donor cells and mouse graft recipients to circumvent the need for immunosuppression. Through multiple behavioral tasks, previously shown to be useful in assessing the 6-OHDA MFB lesion in mice (Heuer et al., 2012), we found impairments on the rotarod and balance beam tasks, with a significant rotational bias over unlesioned, control mice both spontaneously and in response to amphetamine. Evaluation of motor (Heuer et al., 2013), cognitive (Heuer et al., 2013), and dyskinetic behaviors (Smith et al., 2012b) have been reported following transplantation of primary mouse VM derived DAergic cells. Whilst there have been some direct comparisons of primary versus pluripotent stem cell derived DAergic cells transplanted in the 6-OHDA model (Grealish et al., 2014), this study is distinct in that it uses species compatible donor cells and alongside assessment of functional recovery also evaluates both L-DOPA and amphetamine-induced AIMs. We observed modest functional recovery which was mirrored in both transplant groups. The most sensitive tests for capturing modest effects in mice were the rotarod and amphetamine induced rotation. Of note, the EpiSC transplant group, and not the primary VM transplant group, showed a degree of recovery on the rotarod at 16 weeks post-transplantation, with latency to fall no longer significantly different from that of the unlesioned control group. Importantly, the majority of literature on the 6-OHDA lesion model with subsequent dopamine precursor transplantation to assess functional recovery is in the rat (Yurek and Fletcher-Turner, 2004; Rodriguez-Gomez et al., 2007; Peng and Copray, 2016; Bjorklund and Dunnett, 2019). There needs to be further optimisation of transplantation procedures and behavioral tests for functional readout in the mouse which will afford greater flexibility to explore genetic influences without the complications of immunosuppression.

Graft induced dyskinesias were first identified by Freed at al following transplantation of human fetal VM tissue into PD patients (Freed et al., 2001) and subsequently observed following another US trial as well as retrospectively in a European trial (Hagell et al., 2002; Olanow et al., 2003). They can occur despite a beneficial reduction in LIDs seen following transplantation in experimental models (Carlsson et al., 2009; Lane et al., 2009a). Since identification of this side effect there has been much research to understand GIDs, so that they may mitigated in future trials. Experimentally, behaviors aligned to GIDs can be triggered in grafted 6-OHDA lesioned rats by administration of amphetamine (Lane and Smith, 2010). Amphetamine-induced AIMs have been found to be associated with L-DOPA priming, graft composition, graft placement, size, and potentially synaptic plasticity (Carlsson et al., 2006; Lane et al., 2006, 2009a; Carta et al., 2010; Rylander Ottosson and Lane, 2016). We previously generated a model for amphetamine-induced AIMs in the 6-OHDA lesioned, L-DOPA treated mouse demonstrating induction of these behaviors 16 weeks after transplantation of primary mouse VM (Smith et al., 2012b). Here, despite limited functional efficacy and no meaningful reduction in L-DOPA induced AIMs compared to lesion-only controls, amphetamine-induced AIMs were established equally in both transplant groups. Importantly the magnitude of the amphetamine-induced AIMs was significantly less than the L-DOPA induced AIMs, consistent with reports from patients that GID in the majority of cases was mild in severity (Hagell et al., 2002). Induced in both transplant groups equally, it may be that GIDs are an inevitable risk following transplantation regardless of the donor cell source. If transplantation can be optimized to provide reliable and meaningful improvements in motor function and reductions in L-DOPA medication, the benefits could far outweigh the potentially mild graft-induced motor side effects which are significantly less troublesome than LIDs which are an inevitability for the majority of patients under current treatment options. Evidence from other studies suggests that these could be mitigated by selecting patients with well-defined denervation and limited LID development with current medication (Piccini et al., 2005; Lane et al., 2009a; Barker et al., 2017). Importantly, here we present data validating the mouse 6-OHDA model for dyskinesia research following pluripotent stem cell transplantation for the first time.

We report differences in the morphology and cell soma of VM-derived and EpiSC-derived DAergic neurons following transplantation with no differences in the numbers of TH immunopositive cells or their innervation into the host striatum. With respect to functional assessment of the grafts, we present no obvious differences between primary VM and EpiSC transplants on the parameters investigated indicating that pluripotent stem cell sources offer a realistic option for future transplantation clinical trials for PD and given the right conditions and further optimisation they may be able to be utilized as a long-lasting dopamine replacement strategy. Pluripotent stem cell sources have benefits over primary tissue because they side-step some of the key issues intrinsic to fetal tissue: supply, quantity, and consistency. Their production can be scaled up with batch production and they can be potentially grown in bioreactors and banked frozen until required (Nolbrant et al., 2017; Wakeman et al., 2017). Robust differentiation protocols can be quality assessed at critical stages using identification markers of authentic differentiation, and microbiology testing, to ensure acceptable standards are met (Kirkeby et al., 2017).

It will be important in future studies to optimize transplant parameters in order to maximize functional recovery whilst minimizing adverse effects. Strategies may include optimisation of the cellular composition of the donor source, donor cell number, graft location, and delivery method, etc. In order to realize the potential for clinical application of pluripotent stem cells further understanding of their *in vivo* capabilities compared with the gold standard primary VM in an allograft model is required. This would include stringent assessment of function, including evaluation of the potential to produce dyskinesias. Since there is an abundance of mouse and human PSC lines and very few robust rat PSC lines, transplantation of PSCs in an allograft system is most likely in the mouse, as presented here. Allograft studies will be invaluable in enabling us to gain further insight into the differences and similarities in the functional properties of different cell sources following engraftment into the disease host brain.

## Data Availability Statement

The datasets generated for this study are available on request to the corresponding author.

## Ethics Statement

The animal study was reviewed and approved by the Animal Welfare and Research Ethics Board at Cardiff University (Project license 30/3036, awarded by the Home Office).

## Author Contributions

SP, GS, AH, IJ, and CK carried out the experiments. SP, GS, and AH performed analysis of data. SP, GS, AH, CK, SD, and AR designed the experiment. SP, GS, AH, CK, EL, and AR wrote the manuscript. SP, GS, AH, CK, EL, ML, SD, and AR reviewed the manuscript.

## Conflict of Interest

The authors declare that the research was conducted in the absence of any commercial or financial relationships that could be construed as a potential conflict of interest.
